# Post-exercise hypoglycaemia in adults with cystic fibrosis compared to healthy control adults

**DOI:** 10.1016/j.jcte.2026.100456

**Published:** 2026-09-04

**Authors:** Tiffany J. Dwyer, Yuankai Gu, Ruth Dentice, Molly Cocks, Alana Hayes, Helen Jo, Jessica Marouvo, Samantha Nolan, Nicole Taylor, Simone Visser, Veronica Yozghatlian, Katrin Kosbab-Jackson, Sheila Sivam

**Affiliations:** a Discipline of Physiotherapy, Sydney School of Health Sciences, Faculty of Medicine and Health, University of Sydney, Australia; b Department of Respiratory and Sleep Medicine, Royal Prince Alfred Hospital, Australia; c Department of Physiotherapy, Royal Prince Alfred Hospital, Australia; d Department of Endocrinology, Royal Prince Alfred Hospital, Australia; e Central Clinical School, Sydney Medical School, Faculty of Medicine and Health, University of Sydney, Australia

**Keywords:** CGM, Continuous glucose monitor, Diabetes, Glycaemic control, Elexacaftor/tezacaftor/ivacaftor

## Abstract

**Background:**

People with cystic fibrosis-related diabetes (CFRD) are at risk of hypoglycaemia related to insulin therapy; and hypoglycaemia is also prevalent in those without CFRD. Although people with other forms of diabetes mellitus are at risk of hypoglycaemia within 48 h after exercise, this has not been objectively measured in people with CF. This study investigated the prevalence of post-exercise hypoglycaemia in adults with CF (pre- and post-commencement of elexacaftor/tezacaftor/ivacaftor (ETI)), compared to healthy controls.

**Methods:**

Participants completed a 20-min supervised, moderate-intensity cycling session and wore a continuous flash glucose sensor. The prevalence of hypoglycaemia was defined as the number of participants and percentage of time glucose levels were < 3.9 mmol/L and < 3.0 mmol/L within the 48 h after exercise.

**Results:**

15 adults with CF pre-ETI (abnormal glucose tolerance (impaired glucose tolerance, *n* = 2, or CFRD, *n* = 5)), 32 adults with CF post-ETI (21 abnormal glucose tolerance (impaired glucose tolerance, *n* = 10, or CFRD, *n* = 11)) and 15 healthy controls completed the study. There were no significant differences in the prevalence or duration of hypoglycaemia post-exercise between the CF and control groups; and no change after commencing ETI. There was also no significant increase in the prevalence or duration of hypoglycaemia for people with CF with abnormal glucose tolerance when comparing the 48 h post-exercise period to the 48 h period with the least exercise.

**Conclusions:**

The absence of any increase in significant hypoglycaemia in the post-exercise period should be reassuring for people with CF who have abnormal glucose tolerance, whether prescribed ETI or not, and their clinicians.

## Highlights

• No difference in post-exercise hypoglycaemia between CF and healthy control groups.
• No increase in post-exercise hypoglycaemia in abnormal glucose tolerance compared to periods of no/minimal exercise.
• No increase in post-exercise hypoglycaemia with elexacaftor/tezacaftor/ivacaftor.

## Introduction

Cystic fibrosis (CF) is associated with complex multi-organ disease [1] and is estimated to affect more than 180,000 people worldwide, with increasing awareness of CF also occurring in people with non-European ancestry [2]. Dysglycaemia and CF-related diabetes (CFRD) are among the most common comorbidities for people with CF, increasing in prevalence with advancing age [[3], [4]]- with the most recent data registry reports recording a prevalence of impaired glucose tolerance (IGT) at 12.0% [4] and CFRD at 28.0–29.3% among adults with CF [3], [4], [5]. People with CFRD are at risk of hypoglycaemia related to insulin therapy [6], [7]. Hypoglycaemia is also prevalent in people with CF in the absence of established diabetes, detected during oral glucose tolerance tests (OGTT) and in free-living conditions with continuous glucose monitoring (CGM), up to 59% and 69% respectively [8]. It is often reactive hypoglycaemia, though fasting hypoglycaemia attributed to undernourishment with underlying inflammation or acute infection is a concern [9]. Pathophysiology is multifactorial and not well-understood, though likely due to a delayed first phase insulin response and also impaired counter regulation with glucagon [7].

Early studies investigating the effect of the CFTR modulator elexacaftor/tezacaftor/ivacaftor (ETI) on glucose metabolism have shown mixed results [10], potentially due to small sample sizes and differences in included cohorts. Some studies showed commencement of ETI improved blood glucose, measured as glycated haemoglobin (HbA1c) and increased time in target range with CGM [10], though ETI does not appear to improve OGTT glucose excursion or insulin secretion [11]. The effect of ETI on hypoglycaemia remains unclear due to only a few studies with small sample sizes (median *n* = 11) [12], [13], [14], [15], [16], [17], [18], though earlier generations of CFTR modulators increased hypoglycaemia for some people with CFRD [19].

Exercise is a recommended adjunct to pharmacological and dietetic management for people with type 1 (T1DM) and type 2 diabetes mellitus (T2DM) to facilitate improved glycaemic control [20]. Exercise is also recommended for all people with CF [21], including those with CFRD [9], [22], though there is no published study investigating the effect of exercise or physical activity on glycaemic control in people with CFRD. Despite the undoubted benefits of exercise in managing T1DM and T2DM, there is a risk of hypoglycaemia within 48 h after exercise [23]. To date, however, little is known about the presence of post-exercise hypoglycaemia in people with CF.

In a retrospective recall study investigating severe adverse events with exercise, only 2/256 people with CF reported exercise-related hypoglycaemia [24]. This is likely underestimated, however, as participants were not specifically asked about hypoglycaemia or common symptoms associated with hypoglycaemia, they may not have considered it “severe”, and it was only reported by participants in response to “any other problem?”. In addition, hypoglycaemia is frequently asymptomatic for people with CF [8], so some participants may have been unaware of post-exercise hypoglycaemia. The only robust and valid way to assess post-exercise hypoglycaemia prevalence, therefore, is to prospectively measure glucose levels via CGM following an exercise session. This allows for objective measurement of glucose levels, including nocturnally, and avoids under-reporting from asymptomatic hypoglycaemia. Considering people with CF have increased prevalence of hypoglycaemia, and that hypoglycaemia may be exacerbated by ETI, it is therefore important to investigate, via continuous objective measurement, the prevalence of post-exercise hypoglycaemia in people with CF both before and after commencement of ETI.

The aim of this study was to investigate hypoglycaemia, using flash glucose monitoring, after moderate-intensity aerobic exercise in adults with CF pre-ETI, adults with CF at least six months post-commencement of ETI, and healthy control adults. The research questions were: is there a difference in prevalence and amount of time in post-exercise hypoglycaemia between:

1. CF groups compared to the healthy control group?
2. CF group with normal glucose tolerance (NGT) compared to the CF group with abnormal glucose tolerance (AGT)?
3. CF group pre-ETI compared to post-commencement of ETI?

## Materials and methods

### Study design

An observational, single-centre study was conducted over 10 days, during which participants maintained their usual physical activity, diet and medication regimens. On Day1, participants installed the FreeStyle LibreLink App (Abbott, USA) on their mobile phone and were provided a continuous flash glucose sensor (FreeStyle Libre 2, Abbott, USA), which recorded the interstitial glucose every 15 min. Participants scanned the sensor with their phone at least every eight hours, transferring the CGM sensor glucose data to the App. On one occasion, between Day4-Day8, participants attended the hospital for lung function testing [25], [26], a dual-energy X-ray absorptiometry (DEXA) body composition scan (Lunar Prodigy, GE HealthCare, USA) and a supervised exercise session.

Research procedures were approved by the Royal Prince Alfred Hospital (RPAH) Human Research Ethics Committee and participants provided written, informed consent prior to data collection. The trial was prospectively registered with the Australian and New Zealand Clinical Trials Registry (#ACTRN12622000385741).

### Participants

Participants with CF were recruited from the RPAH adult CF clinic and healthy controls were recruited via posters displayed in RPAH clinic waiting rooms and University of Sydney notice boards. Participants with CF, who had not commenced ETI before the first 10-day data collection period, repeated the study at least six months after commencing ETI. Potential participants with CF were eligible if they: had a confirmed diagnosis of CF (genetic testing and/or previous positive sweat test); were aged at least 17 years; deemed clinically-stable by their treating physician, including no non-routine antibiotics in the previous 14 days; and eligible for ETI prescription on the Australian Pharmaceutical Benefits Scheme. Healthy control participants were eligible if they were aged at least 17 years and had no history of cardiovascular, neuromuscular, endocrine or respiratory disease. Potential participants were excluded if they: were unable to perform moderate-intensity exercise; were pregnant or lactating; were colonised with *Burkholderia cepacia* complex or non-tuberculous mycobacteria; had received a lung transplant; had known hypoglycaemia unawareness; were unable to use a CGM device; or did not have a mobile phone compatible with the CGM device.

### Intervention

The exercise session, between Day4-Day8 of the trial, was performed on a stationary cycle (Spirit Medical MU100 Rehabilitation Upright Bike, Spirit Medical System Group, Canada) and supervised by a physiotherapist, monitoring the participant's pulse rate, oxygen saturation and symptoms of breathlessness [27] and fatigue [28]. The workload for the 20-min moderate-intensity cycling exercise was individually tailored to achieve 60–70% of the participant's estimated peak heart rate (210- (0.65 x age,years)) [29] and reported breathlessness of moderate-somewhat severe (3–4 on the Borg dypsnoea scale), as per pulmonary rehabilitation guidelines [30]. Participants with CFRD were instructed to administer their usual prandial insulin, including any routine dose adjustments before exercise; and were provided 15–30 g of fast-acting carbohydrate if their BGL was <5.6 mmol/L before starting exercise. Participants were observed for one hour after completion of the exercise session and provided with a hypoglycaemia kit (fast and long-acting carbohydrate) if they showed any sign of hypoglycaemia.

### Outcomes

Participants with CF were classified by the CF endocrinologist as NGT, IGT or CFRD (with IGT and CFRD combined as AGT), referencing their most recent OGTT [31] performed as part of their annual review. The research team accessed the CGM data via the LibreView (Abbott, USA) cloud-based platform. Comparisons were made between the 48 h period after the supervised exercise session to the final 48 h period where no (or the least amount of) exercise was performed in a four-day window of the 10 days of data collection (to minimise any potential carryover effect on hypoglycaemia from exercise performed in the preceding 48 h). The primary outcome was the prevalence and percentage of time in mild hypoglycaemia (CGM glucose levels <3.9 mmol/L) [8] within the 48 h after the supervised exercise session. Secondary outcomes were the: prevalence and percentage of time in significant hypoglycaemia (CGM glucose levels <3.0 mmol/L) [8] within the 48 h after the supervised exercise session; number of participants requiring treatment for hypoglycaemia within the 1 h after the supervised exercise session; value and time post-exercise for the nadir glucose in those with hypoglycaemia; measures of fat-free mass and visceral adipose tissue from the DEXA body composition scan, total fat-free mass in kilograms, and visceral adipose tissue mass in grams. Descriptive measures of CGM sensor active time and glycaemic control in the final seven days of data collection were also reported: average glucose in mmol/L; peak glucose in mmol/L; %time in target range, 3.9–10.0 mmol/L; %time in hyperglycaemia, >10.0 mmol/L; %time in hypoglycaemia, <3.9 mmol/L and < 3.0 mmol/L; and glycaemic variability, measured as the standard deviation of glucose divided by the average glucose, reported as a percentage.

### Data analysis

Descriptive statistics were reported as mean and standard deviation if normally-distributed or median and interquartile range if not normally-distributed. The primary and secondary outcome measures, prevalence of post-exercise hypoglycaemia <3.9 mmol/L and < 3.0 mmol/L within the 48 h after the supervised exercise session, were reported as the percentage of the total sample and compared between the three groups (CF compared to healthy controls; CF-NGT compared to CF-AGT; CF-pre-ETI compared to CF-post-ETI) using chi-square tests. Differences between the three groups in the percentage of time in post-exercise hypoglycaemia <3.9 mmol/L and < 3.0 mmol/L within the 48 h after the supervised exercise session were assessed by unpaired Mann-Whitney *U* tests. Comparisons between groups for the secondary outcome measure of the number of participants requiring treatment for hypoglycaemia in the 1 h after the exercise session were performed using chi-square tests. Associations between the primary outcome measures and the secondary measures of body composition were analysed with Spearman's rank correlation coefficients. A *p*-value of <0.05 was considered statistically significant for all analyses.

There are no studies using CGM data to assess post-exercise hypoglycaemia and glycaemic control in people with CF, so the intended sample size for this study is based from a study, which reported the 3-month repeatability of CGM measures in CF adults to have a mean change in average glucose of 8.5 mg/dL (SD 10.6 mg/dL), corresponding to 0.5 and 0.6 mmol/L respectively [16], [32]. With a two-tailed significance level of α = 0.05 and power at 80%, a sample size of 15 participants is required (G*Power v 3.1.9.6). We therefore aimed to recruit 15 participants per each of the CF and healthy control groups. This is likely a conservative estimate, as it would be expected to have less variability in glycaemic control during the 10-day data collection period of this study compared to the 3-month interval in the previous study. Other studies found significant differences with smaller sample sizes when using CGM to measure glycaemic control following CFTR modulators in CF adults (*n* = 14 with CFRD and *n* = 9 with NGT) [16] and different types of exercise in people with T1DM (*n* = 12) [33].

## Results

Thirty-six adults with CF and 15 healthy controls were recruited; nine participants with CF short of our intended sample size, but recruitment options were exhausted within our clinic. Three participants with CF dropped out of the study before CGM insertion, with no data collected, one dropped out after pre-ETI data collection and 14 participated both pre-ETI and post-ETI. In the post-ETI cohort, participants commenced ETI 214 ± 61 days prior to data collection. The healthy control adults were similarly matched to the CF participants in terms of age, sex and BMI (Table 1 and supplementary Table S1 for the 14 participants both pre- and post-ETI). In the pre-ETI cohort, 7/15 were classified as AGT (IGT or CFRD) and in the post-ETI cohort, 21/32 were classified as AGT (supplementary Table S2 for IGT and CFRD group data). Of those diagnosed with CFRD (*n* = 5 pre-ETI and *n* = 11 post-ETI), all were managed with multiple daily injections of insulin (basal-bolus (*n* = 6) or bolus only (*n* = 3)) except for two participants pre-ETI and one participant post-ETI, who were managed with diet-control only (one of the pre-ETI diet-control participants changed to bolus only insulin before post-ETI data collection). No participant had a standard or hybrid closed-loop insulin pump.

**Table 1:** Participant characteristics.

	Control	CF			CF		
		All	NGT	AGT	All	NGT	AGT
**Number, *n***	15	15	8	7	32	11	21
**Age, *yr***	36.4 ± 15.3	36.9 ± 13.6	39.1 ± 17.7	34.4 ± 7.6	37.2 ± 13.0	40.7 ± 14.9	35.4 ± 11.8
**Sex, *F:M***	7: 8	4: 11	2: 6	2: 5	13: 19	4: 7	9: 12
**BMI, *kg/m***^***2***^	24.9 ± 4.3	24.3 ± 3.2	24.3 ± 3.5	24.3 ± 3.0	23.8 ± 3.8	24.0 ± 4.2	23.8 ± 3.8
**FEV**_**1**_**, *%pp***	100 ± 11	82 ± 17	87 ± 15	77 ± 20	85 ± 20	88 ± 14	84 ± 22
**FVC, *%pp***	100 ± 14	99 ± 13	105 ± 10	91 ± 12	100 ± 15	101 ± 12	100 ± 17
**Genotype, *n***							
Homozygous F508del		4	1	3	17	3	14
Heterozygous F508del		11	7	4	15	8	7
**Panc. status, *PI:PS***		14: 1	7: 1	7: 0	28: 4	8: 3	20: 1
**CFTRm (before ETI), *n***							
Ivacaftor		2	0	2	3	1	2
Lumacaftor/ivacaftor		2	0	2	7	1	6
Tezacaftor/ivacaftor		2	1	1	8	2	6
**DEXA body composition scan**							
Number, *n*	14				22	9	13
FFM, *kg*	51.4 ± 11.9				50.9 ± 10.7	52.0 ± 11.4	50.1 ± 10.6
VAT mass, *g*	444 ± 426				408 ± 447	424 ± 591	398 ± 358

Data are presented as number or mean ± SD as appropriate. Glucose tolerance classification from most recent OGTT. DEXA body composition scans were unable to be performed pre-ETI for the participants with CF. Fourteen participants with CF participated both at baseline (pre-ETI) and follow-up (post-ETI). AGT: abnormal glucose tolerance (diagnosed as impaired glucose tolerance or CF-related diabetes); BMI: body mass index; CF: cystic fibrosis; CFTRm (before ETI): cystic fibrosis transmembrane conductance regulator modulator prescribed prior to starting ETI (all participants were taking ETI at the follow-up, post-ETI visit); DEXA: dual energy x-ray absorptiometry; ETI: elexacaftor/tezacaftor/ivacaftor; F: female; FEV_1_: forced expiratory volume in 1 s; FFM: fat free mass; FVC: forced vital capacity; g: gram; kg: kilogram; m: metre; M: male; n: number; NGT: normal glucose tolerance; OGTT: oral glucose tolerance test; Panc. status: pancreatic status; PI: pancreatic insufficient; PS: pancreatic sufficient; %pp: percent of predicted value [26]; SD: standard deviation; VAT: visceral adipose tissue; yr: years.

Participants had good adherence with CGM sensor scanning with median sensor active time 92.4% (83.0,97.5). The CGM glycaemic control data for the final seven days of data collection is found in Table 2 (supplementary Table S3 for IGT and CFRD group data). The supervised exercise session was well-tailored to the target moderate intensity with mean pulse rate 66% ± 9 of estimated peak (Table 3 and supplementary Table S4 for the 14 participants both pre- and post-ETI). In the four-day window with the least amount of exercise, the majority of participants performed no or less than 20 min of moderate-intensity aerobic exercise (CF group pre-ETI *n* = 8/15, CF group post-ETI *n* = 18/32, control group *n* = 8/15); and few performed 20 min or more of moderate-intensity aerobic exercise during the 48 h least exercise comparison period to the 48 h post-supervised exercise period (CF group pre-ETI *n* = 1/15, CF group post-ETI *n* = 4/32, control group *n* = 5/15). The comparison 48 h period with the least amount of exercise was mainly captured before the supervised exercise session (CF group pre-ETI *n* = 10/15, CF group post-ETI *n* = 25/32, control group *n* = 11/15).

**Table 2:** Continuous glucose monitoring results.

		Baseline, pre-ETI			Follow-up, post-ETI		
	Control	CF			CF		
		All	NGT	AGT	All	NGT	AGT
Number, *n*	15	15	8	7	32	11	21
Average sensor glucose, *mmol/L*	5.5 ± 0.4	6.5 ± 0.8	6.1 ± 0.6	7.0 ± 0.7	7.1 ± 1.2	6.3 ± 0.5	7.5 ± 1.3
Minimum sensor glucose, *mmol/L*	3.8 ± 0.7	3.5 ± 0.6	3.6 ± 0.6	3.4 ± 0.7	3.5 ± 0.7	3.7 ± 0.4	3.4 ± 0.8
Maximum sensor glucose, *mmol/L*	9.2 ± 1.4	13.2 ± 3.1	11.4 ± 2.6	15.2 ± 2.3	15.2 ± 4.8	11.4 ± 1.8	17.2 ± 4.7
Time in range (3.9–10.0 mmol/L), *%*	99.4 (98.8,100.0)	94.7 (90.8,98.7)	98.6 (94.7,99.2)	92.4 (73.2,94.7)	94.7 (76.8,97.7)	98.0 (96.0,99.4)	87.2 (70.7,95.9)
Time < 3.9 mmol/L, *%*	0.6 (0.0,1.2)	0.6 (0.2,1.3)	0.2 (0.0,0.8)	0.9 (0.2,6.4)	0.5 (0.0,2.1)	0.2 (0.0,1.1)	0.8 (0.0,2.6)
Time < 3.0 mmol/L, *%*	0.0 (0.0,0.0)	0.0 (0.0,0.0)	0.0 (0.0,0.0)	0.0 (0.0,0.2)	0.0 (0.0,0.2)	0.0 (0.0,0.0)	0.0 (0.0,0.5)
Time > 10.0 mmol/L, *%*	0.0 (0.0,0.0)	5.2 (1.0,8.9)	1.1 (0.0,5.3)	7.4 (5.2,18.1)	4.7 (1.8,21.3)	1.3 (0.0,2.6)	12.3 (3.4,28.5)
Standard deviation sensor glucose, *mmol/L*	0.8 ± 0.2	1.7 ± 0.7	1.2 ± 0.4	2.2 ± 0.7	2.0 ± 1.1	1.2 ± 0.2	2.5 ± 1.1
Co-efficient of variation, *SD/X̄*	0.14 ± 0.03	0.25 ± 0.08	0.20 ± 0.06	0.31 ± 0.08	0.27 ± 0.10	0.19 ± 0.03	0.32 ± 0.10
Time sensor active, *%*	89.9 (74.9,96.0)	93.3 (89.7,98.7)	94.2 (82.7,99.0)	92.9 (91.1,98.4)	91.7 (82.7,97.4)	92.9 (83.3,97.5)	91.1 (81.4,97.5)

Data are presented as number, mean ± SD or median (IQR) as appropriate for the Continuous Glucose Monitor (CGM) results summarised from final seven days of data collection. Glucose tolerance classification from most recent OGTT. Fourteen participants with CF participated both at baseline (pre-ETI) and follow-up (post-ETI). AGT: abnormal glucose tolerance (diagnosed as impaired glucose tolerance or CF-related diabetes); CF: cystic fibrosis; ETI: elexacaftor/tezacaftor/ivacaftor; IQR: interquartile range; n: number; NGT: normal glucose tolerance; OGTT: oral glucose tolerance test; SD: standard deviation; $\bar{X}$: mean.

**Table 3:** Supervised exercise session.

		Baseline, pre-ETI			Follow-up, post-ETI		
	Control	CF			CF		
		All	NGT	AGT	All	NGT	AGT
**Number, *n***	15	15	8	7	32	11	21
**Watts**	73 ± 25	62 ± 13	67 ± 12	58 ± 14	57 ± 19	64 ± 22	55 ± 18
**HR, *bpm***	123 ± 12	117 ± 11	114 ± 14	119 ± 6	124 ± 23	122 ± 26	125 ± 21
**HR, *%pk***	66 ± 5	63 ± 6	62 ± 8	64 ± 3	67 ± 11	67 ± 14	67 ± 10
**SpO**_**2**_**, *%***	97 ± 1	96 ± 2	95 ± 2	96 ± 2	97 ± 1	96 ± 1	97 ± 1
**Dyspnoea**	1.4 ± 0.7	2.2 ± 0.9	1.9 ± 1.0	2.5 ± 0.6	2.3 ± 1.5	2.4 ± 1.0	2.2 ± 1.7
**RPE**	1.8 ± 1.5	2.5 ± 0.9	2.4 ± 1.0	2.7 ± 0.9	2.7 ± 1.4	2.9 ± 1.4	2.7 ± 1.5
**Sensor glucose**							
Pre exercise	5.4 ± 0.8	6.4 ± 1.0	6.6 ± 1.1	6.1 ± 0.9	7.2 ± 2.5	6.2 ± 1.4	7.8 ± 2.8
Post exercise	5.1 ± 0.6	5.9 ± 1.7	5.9 ± 1.5	6.0 ± 2.0	6.9 ± 2.6	6.5 ± 1.2	7.2 ± 3.2

Data are presented as number or mean ± SD as appropriate. Glucose tolerance classification from most recent OGTT. Sensor glucose as recorded by CGM sensor immediately pre and post 20 min exercise session. Fourteen participants with CF participated both at baseline (pre-ETI) and follow-up (post-ETI). AGT: abnormal glucose tolerance (diagnosed as impaired glucose tolerance or CF-related diabetes); bpm: beats per minute; CF: cystic fibrosis; ETI: elexacaftor/tezacaftor/ivacaftor; HR: heart rate (during 20 min exercise); n: number; NGT: normal glucose tolerance; OGTT: oral glucose tolerance test; %pk: percentage of estimated peak heart rate [29]; RPE: rating of perceived exertion; SD: standard deviation; SpO_2_: peripheral oxygen saturation.

### Prevalence and percentage of time in hypoglycaemia post-exercise

There was no significant difference in the prevalence of mild hypoglycaemia (sensor glucose <3.9 mmol/L) in the 48 h after the supervised exercise session between the CF groups and the control group (CF group pre-ETI *n* = 6/15, CF group post-ETI *n* = 9/32, control group *n* = 5/15). There was no significant difference in the percentage of time in mild hypoglycaemia (sensor glucose <3.9 mmol/L) in the 48 h after the supervised exercise session between the CF groups and the control group (CF group pre-ETI 0.0% (0.0,1.1), CF group post-ETI 0.0% (0.0,0.9), control group 0.0% (0.0,1.1)). There was also no significant difference in the prevalence or percentage of time in significant hypoglycaemia (sensor glucose <3.0 mmol/L) in the 48 h after the supervised exercise session between the CF groups and the control group (CF group pre-ETI *n* = 1/15, CF group post-ETI *n* = 4/32, control group *n* = 0/15; and all groups 0.0% (0.0,0.0) respectively).

There was a significantly higher prevalence of mild hypoglycaemia (sensor glucose <3.9 mmol/L) in the 48 h after the supervised exercise session for CF participants with AGT (IGT or CFRD) compared to those with NGT both pre- and post-ETI (pre-ETI: AGT *n* = 5/7 v NGT *n* = 1/8; *p* < 0.05; post-ETI: AGT *n* = 9/21 v NGT *n* = 0/11, *p* < 0.05). There was also significantly longer time in mild hypoglycaemia (sensor glucose <3.9 mmol/L) in the 48 h after the supervised exercise session for CF participants with AGT (IGT or CFRD) compared to those with NGT both pre- and post-ETI (pre-ETI: AGT 0.5% (0.0,3.1) v NGT 0.0% (0.0,0.0), *p* < 0.05; post-ETI: AGT 0.0% (0.0,1.5) v NGT 0.0% (0.0,0.0), *p* < 0.05). There was, however, no significant difference in the prevalence or percentage of time in significant hypoglycaemia (sensor glucose <3.0 mmol/L) in the 48 h after the supervised exercise session between the CF participants with AGT (IGT or CFRD) compared to those with NGT both pre- and post-ETI (pre-ETI: AGT *n* = 1/7, NGT *n* = 0/8; post-ETI: AGT *n* = 4/21 v NGT *n* = 0/11; and all groups 0.0% (0.0,0.0) respectively).

When comparing the prevalence of mild hypoglycaemia (sensor glucose <3.9 mmol/L) for the CF participants with AGT (IGT or CFRD) between the 48 h period after the supervised exercise session and the 48 h period with the least amount of exercise, there were no significant differences both pre- and post-ETI (pre-ETI: 48 h least exercise *n* = 2/7, 48 h post-supervised exercise *n* = 5/7; post-ETI: 48 h least exercise *n* = 12/21, 48 h post-supervised exercise *n* = 9/21). There was also no significant difference in the percentage of time in mild hyperglycaemia (sensor glucose <3.9 mmol/L) for the CF participants with AGT (IGT or CFRD) between the two 48 h periods (pre-ETI: 48 h least exercise 0.0% (0.0,1.1), 48 h post-supervised exercise 0.5% (0.0,3.1); post-ETI: 48 h least exercise 0.5% (0.0,2.1), 48 h post-supervised exercise 0.0% (0.0,1.5)).

For the CF participants who completed data collection both pre- and post-ETI, there was no significant difference in the prevalence of mild hypoglycaemia (sensor glucose <3.9 mmol/L) or significant hypoglycaemia (sensor glucose <3.0 mmol/L) in the 48 h after the supervised exercise session between the two periods (pre-ETI *n* = 6/14, post-ETI *n* = 3/14; and pre-ETI *n* = 1/14, post-ETI *n* = 1/14 respectively). There was also no significant difference in the percentage of time in mild hypoglycaemia (sensor glucose <3.9 mmol/L) or significant hypoglycaemia (sensor glucose <3.0 mmol/L) in the 48 h after the supervised exercise session between the two periods (pre-ETI 0.0% (0.0,1.2), post-ETI 0.0% (0.0,0.3); and 0.0% (0.0,0.0) both time periods respectively).

No healthy control participant and no CF participant pre-ETI required treatment for hypoglycaemia in the 1 h after the exercise session. Two participants with CF post-ETI, however, did require a fast-acting carbohydrate snack within 5 min of finishing the exercise session (*n* = 1 IGT and *n* = 1 CFRD), though there were no significant differences between groups. Neither of those participants completed the study pre-ETI.

Among the participants who experienced mild hypoglycaemia (sensor glucose <3.9 mmol/L) in the 48 h after the supervised exercise session, the nadir value was similar between groups, though varied when it occurred (Table 4 and supplementary Table S5 for IGT and CFRD group data). For control participants with mild hypoglycaemia in the 48 h after the supervised exercise period, the nadir occurred most frequently in the second 24 h period (*n* = 4/5), which was similar for the CF group pre-ETI (*n* = 4/6), however for the CF group post-ETI the nadir most frequently occurred in the first 24 h period (*n* = 7/9).

**Table 4:** Nadir glucose and time post-exercise for participants with post-exercise hypoglycaemia.

		Baseline, pre-ETI			Follow-up, post-ETI		
	Control	CF			CF		
		All	NGT	AGT	All	NGT	AGT
**Hypo, *Y: N***	5: 10	6: 9	1: 7	5: 2	9: 23	0: 11	9: 12
**Nadir glucose, *mmol/L***	3.4 ± 0.3	3.4 ± 0.4	3.3	3.5 ± 0.4	3.1 ± 0.4	–	3.1 ± 0.4
**Time post-exercise, *hr***	38.2 (15.8,42.8)	33.5 (10.6,41.9)	32.3	34.7 (7.3,42.9)	12.5 (5.6,24.0)	–	12.5 (5.6,24.0)
0-12 h, *n*	1	1	0	1	4	–	4
12-24 h, *n*	0	1	0	1	3	–	3
24-36 h, *n*	1	2	1	1	1	–	1
36-48 h, *n*	3	2	0	2	1	–	1

Data are presented as number, mean ± SD or median (IQR) as appropriate for the participants in each group with hypoglycaemia (sensor glucose <3.9 mmol/L) in the 48 h after the supervised exercise session. Glucose tolerance classification from most recent OGTT. AGT: abnormal glucose tolerance (diagnosed as impaired glucose tolerance or CF-related diabetes); CF: cystic fibrosis; ETI: elexacaftor/tezacaftor/ivacaftor; h: hour; hypo: hypoglycaemia (sensor glucose <3.9 mmol/L) present or not in the 48 h post-exercise; IQR: interquartile range; n: number; N: no; NGT: normal glucose tolerance; OGTT: oral glucose tolerance test; SD: standard deviation; Y: yes.

### Associations between body composition and post-exercise hypoglycaemia

There was no significant association, measured with Spearman's *rho*, between either FFM or VAT mass and the percentage of time in mild hypoglycaemia (sensor glucose <3.9 mmol/L) in the 48 h after the supervised exercise session for any of the groups (CF, CF-NGT, CF-AGT, control), though there was a trend for the CF group between VAT mass and time in mild hypoglycaemia (*rho* = 0.36, *p* = 0.1).

## Discussion

This is the first study to objectively measure the prevalence of post-exercise hypoglycaemia in people with CF, both before and after commencement of ETI, and compared to healthy controls. The main findings were that there was no difference in the prevalence or duration of mild or significant hypoglycaemia in the 48 h post 20-min of moderate-intensity cycling exercise between the CF and control groups, and also no change after commencing ETI. Although the prevalence of mild hypoglycaemia in the 48 h post-exercise was more prevalent (and of longer duration) in people with CF with AGT (IGT or CFRD) compared to those with NGT, this likely reflects their baseline increased hypoglycaemia, as there were no significant differences in the prevalence or duration of mild hypoglycaemia when comparing the 48 h post-exercise period to the 48 h period with the least exercise. It should also be noted that episodes of post-exercise hypoglycaemia were mild and brief; and the absence of any increase in significant hypoglycaemia in the post-exercise period should be reassuring for people with CF with IGT or CFRD, whether prescribed ETI or not, and their clinicians.

The prevalence of post-exercise mild hypoglycaemia (sensor glucose <3.9 mmol/L) in our study (40% CF pre-ETI, 28% CF post-ETI and 33% healthy controls) was similar to studies reporting CGM during free-living conditions in children and young adults with CF and healthy controls (30–38% and 10–20% respectively) [34], [35], as well as two large studies in adolescents [36] and adults [37] with T1DM (14% and 47% on exercise days respectively). The time in post-exercise mild hypoglycaemia (sensor glucose <3.9 mmol/L) in our study (all groups median 0.0–0.5%) was lower than that reported in studies of CGM during free-living conditions in children and young adults with CF and healthy controls (2.5–4.0% and 1.6–1.8% respectively) [34], [38], the largest study of healthy controls aged 7-80 yr at 1.1% [39], and adolescents [36] and adults [37] with T1DM (1.1–1.9% and 3.1% on exercise days respectively). Importantly, there was no significant increase in the prevalence or duration of significant hypoglycaemia (sensor glucose <3.0 mmol/L) in the 48 h period post-exercise for any group. This is in contrast to adults with T1DM, which have both increased prevalence and duration of significant hypoglycaemia on exercise days compared to sedentary days [37].

In people with T1DM, nadir hypoglycaemia typically occurs 8-16 h after finishing exercise [36], though can occur up to 48 h afterwards [23]. In our study, however, among the healthy controls and adults with CF pre-ETI, who experienced hypoglycaemia within 48 h of the exercise session, the nadir occurred in the first 24 h for only 20% and 32% respectively. In the post-ETI cohort, the nadir occurred in the first 24 h for 78% of participants, which matches the pattern for people with T1DM. In our small dataset without physiological measures, such as insulin secretion or counter-regulatory hormone profiles, we are unable to explain why these apparent differences occurred. Due to the wide individual variability in glycaemic responses, personalised hypoglycaemia management plans for people with CF are warranted.

Most previous studies reporting hypoglycaemia, measured by CGM, before and after ETI initiation in children and adults with CF have shown minimal change in the time in hypoglycaemia (1.3% decrease to 2.0% increase) [13], [14], [15], [16], [17], [18], though one study reported a 12.7% increase [12]. Our study also found no increase in the prevalence or duration of hypoglycaemia in the 48 h post-exercise after ETI initiation, suggesting for most people that there is no increased risk of hypoglycaemia on ETI.

Some limitations of our study are that we did not measure HbA1c or perform OGTT to confirm NGT in the healthy controls, though the 7-day median (IQR) time in range was 99.4% (98.8,100.0) and the lowest was 92.1%, corresponding to an estimated HbA1c ≤5.1% [40]. We did not do finger prick checks of blood glucose levels to confirm accuracy of hypoglycaemic events detected by the CGM, however the flash glucose sensor used in our study (FreeStyle Libre 2) has very good accuracy compared to plasma venous blood glucose in the hypoglycaemia range (>94% of readings within 0.8 mmol/L), over the standard 14-day sensor wear time [41] .There are also potential confounders during the post-exercise monitoring period, as there was no standardised diet, insulin management or time of day for the exercise session, meaning that factors other than just the exercise session may have been attributable to the identified hypoglycaemic events, plus some participants performed other exercise during the 10-day study period. This was, however, purposefully designed to better represent “free-living” conditions and improve clinical relevance. In addition, as the CGM readings were not blinded from participants, it is possible that early detection of decreasing glucose values prompted response to minimise hypoglycaemic events. We only met 80% of our intended sample size for participants with CF and the study recruited from a single CF centre, though the participants had similar lung function, BMI and genotype to that reported in the Australian CF data registry [4], and the sample size of 32 participants with CF post-ETI is larger than all previous studies reporting hypoglycaemia post-ETI (*n* = 6 to 23).

Future research could investigate post-exercise hypoglycaemia following longer durations, intensities (e.g. high-intensity interval training) and types of exercise (e.g. anaerobic and/or resistance training), as studies of exercise in free-living conditions in people with T1DM have shown increased risk of post-exercise hypoglycaemia when the duration of exercise was longer than 60mins/day [36] and significant differences in the fall in glucose levels with different types of activity [37], [42]. The results from our study reinforce the minimal risk of adverse events with exercise for people with CF [24], particularly that there does not appear to be any increase in significant hypoglycaemia following moderate-intensity aerobic exercise, further supporting that exercise should be encouraged for everyone, including those with IGT or CFRD.

## CRediT authorship contribution statement

**Tiffany J. Dwyer:** Writing – review & editing, Writing – original draft, Visualization, Validation, Supervision, Software, Resources, Project administration, Methodology, Investigation, Funding acquisition, Formal analysis, Data curation, Conceptualization. **Yuankai Gu:** Writing – review & editing, Visualization, Validation, Resources, Project administration, Investigation, Formal analysis, Data curation. **Ruth Dentice:** Writing – review & editing, Resources, Investigation. **Molly Cocks:** Writing – review & editing, Resources, Investigation. **Alana Hayes:** Writing – review & editing, Resources. **Helen Jo:** Writing – review & editing, Resources. **Jessica Marouvo:** Writing – review & editing, Resources. **Samantha Nolan:** Writing – review & editing, Resources. **Nicole Taylor:** Writing – review & editing, Resources. **Simone Visser:** Writing – review & editing, Resources. **Veronica Yozghatlian:** Writing – review & editing, Resources. **Katrin Kosbab-Jackson:** Writing – review & editing, Validation, Supervision, Resources, Project administration, Methodology, Investigation, Conceptualization. **Sheila Sivam:** Writing – review & editing, Validation, Supervision, Resources, Project administration, Methodology, Conceptualization.

## Funding

This study was funded by the Australian Cystic Fibrosis Research Trust (sponsored by Mediplast) and supported by the University of Sydney and Royal Prince Alfred Hospital. None of those organisations had any other involvement in this study or its publication.

## Declaration of competing interest

The authors declare that they have no known competing financial interests or personal relationships that could have appeared to influence the work reported in this paper.

## Data Availability

*Will individual participant data be available (including data dictionaries)?* Yes, if approved by the relevant Human Research Ethics Committee. *What data in particular will be shared?* Individual participant data can be shared after de-identification and once approval has been obtained from the relevant Human Research Ethics Committee. *What other documents will be available?* Study protocol. *When will data be available (start and end dates)?* Data will be available for the 7-year data retention period post-publication (as per requirements of the Human Research Ethics Committee) on a case-by-case basis, at the discretion of the co-ordinating principal investigator and relevant Human Research Ethics Committee. *With whom?* Data will be available on a case-by-case basis, at the discretion of the co-ordinating principal investigator and relevant Human Research Ethics Committee. *For what types of analyses?* Data will be available for systematic reviews at the discretion of the relevant Human Research Ethics Committee. *By what mechanism will data be made available?* Data requests should, in the first instance, be addressed to A/Prof Tiffany Dwyer (tiffany.dwyer@sydney.edu.au). Access to data will be subject to approval by the co-ordinating principal investigator and relevant Human Research Ethics Committee.
